# Noncanonical Wnt signaling plays an important role in modulating canonical Wnt-regulated stemness, proliferation and terminal differentiation of hepatic progenitors

**DOI:** 10.18632/oncotarget.15637

**Published:** 2017-02-23

**Authors:** Jiaming Fan, Qiang Wei, Junyi Liao, Yulong Zou, Dongzhe Song, Dongmei Xiong, Chao Ma, Xue Hu, Xiangyang Qu, Liqun Chen, Li Li, Yichun Yu, Xinyi Yu, Zhicai Zhang, Chen Zhao, Zongyue Zeng, Ruyi Zhang, Shujuan Yan, Tingting Wu, Xingye Wu, Yi Shu, Jiayan Lei, Yasha Li, Wenwen Zhang, Rex C. Haydon, Hue H. Luu, Ailong Huang, Tong-Chuan He, Hua Tang

**Affiliations:** ^1^ Key Laboratory of Molecular Biology for Infectious Diseases of The Ministry of Education of China, Institute for Viral Hepatitis, Department of Infectious Diseases, The Second Affiliated Hospital, Chongqing Medical University, Chongqing, China; ^2^ Molecular Oncology Laboratory, Department of Orthopaedic Surgery and Rehabilitation Medicine, The University of Chicago Medical Center, Chicago, IL, USA; ^3^ Ministry of Education Key Laboratory of Diagnostic Medicine, and The Affiliated Hospitals of Chongqing Medical University, Chongqing, China; ^4^ Department of Conservative Dentistry and Endodontics, West China Hospital and West China School of Stomatology, Sichuan University, Chengdu, China; ^5^ Departments of Neurosurgery and Otolaryngology-Head & Neck Surgery, The Affiliated Zhongnan Hospital of Wuhan University, Wuhan, China; ^6^ Department of Biomedical Engineering, School of Bioengineering, Chongqing University, Chongqing, China; ^7^ Department of Emergency Medicine, Beijing Hospital, Beijing, China; ^8^ Department of Orthopaedic Surgery, Union Hospital of Tongji Medical College, Huazhong University of Science & Technology, Wuhan, China; ^9^ Department of Laboratory Medicine and Clinical Diagnostics, The Affiliated Yantai Hospital, Binzhou Medical University, Yantai, China

**Keywords:** Wnt/β-catenin, noncanonical Wnt signaling, liver stem cells, liver cancer, liver regeneration

## Abstract

The liver provides vital metabolic, exocrine and endocrine functions in the body as such pathological conditions of the liver lead to high morbidity and mortality. The liver is highly regenerative and contains facultative stem cells that become activated during injury to replicate to fully recover mass and function. Canonical Wnt/β-catenin signaling plays an important role in regulating the proliferation and differentiation of liver progenitor cells during liver regeneration. However, possible roles of noncanonical Wnts in liver development and regeneration remain undefined. We previously established a reversibly-immortalized hepatic progenitor cell line (iHPx), which retains hepatic differentiation potential. Here, we analyze the expression pattern of the essential components of both canonical and noncanonical Wnt signaling pathways at different postnatal stages of mouse liver tissues and iHPx cells. We find that noncanonical Wnt4, Wnt5a, Wnt9b, Wnt10a and Wnt10b, are highly expressed concordantly with the high levels of canonical Wnts in late stages of liver tissues. Wnt5a, Wnt9b, Wnt10a and Wnt10b are able to antagonize Wnt3a-induced β-catenin/TCF activity, reduce the stemness of iHPx cells, and promote hepatic differentiation of liver progenitors. Stem cell implantation assay demonstrates that Wnt5a, Wnt9b, Wnt10a and Wnt10b can inhibit cell proliferation and promote hepatic differentiation of the iHPx progenitor cells. Our results strongly suggest that noncanonical Wnts may play an important role in fine-tuning Wnt/β-catenin functions during liver development and liver regeneration. Thus, understanding regulatory mechanisms governing proliferation and differentiation of liver progenitor cells may hold great promise to facilitate liver regeneration and/or progenitor cell-based therapies for liver diseases.

## INTRODUCTION

As the largest internal organ and gland in the body, the liver provides vital metabolic, exocrine and endocrine functions, including secretion of several hormones such as insulin-like growth factors, angiotensinogen and thrombopoietin, production of bile, metabolism of dietary compounds, regulation of cholesterol synthesis and transport, urea metabolism, drug detoxification, regulation of glucose levels through glycogen storage, and control of blood homeostasis by secretion of clotting factors and serum proteins such as albumin and apolipoproteins [1–3]. Given the fact the liver plays such critical roles in normal physiological processes, pathological conditions, such as hepatic fibrosis, cirrhosis and hepatitis, and hepatocellular carcinoma, lead to high rates of morbidity and mortality [3]. In fact, liver disease is the fourth leading cause of death among middle-aged adults in the United States [3]. Thus, there is an unmet need in understanding the molecular mechanisms that regulate liver development and liver progenitor cell differentiation.

The liver is one of the most regenerative tissues in the body, capable of fully recovering mass and function after a variety of injuries [4, 5]. The hepatocytes are the principal cell type in the liver and account for ∼70% of the mass of the adult organ. Hepatocytes, along with biliary epithelial cells, are derived from the embryonic endoderm, while the stromal cells, stellate cells, kuppfer cells and blood vessels, are derived from mesoderm [1–3]. The liver has long been speculated to harbor facultative stem cells that can become activated if the injury impairs the ability of the mature cells, especially hepatocytes, to replicate [4, 5]. These facultative stem cells are believed to reside near the portal region of the hepatic lobule in the canal of Hering [4, 5]. After activation, the hepatic stem cells may proliferate and produce ‘oval cells’, intermediate cells that have properties of both bile ducts and hepatocytes, which then would differentiate into functional mature hepatocytes [4, 5]. However, it has been shown that hepatocytes can also be derived from rare cells that reside in the pancreas, bone marrow, and brain [1, 2, 6]. Nonetheless, the liver exhibits extraordinary regenerative potential, which is presumably facilitated by hepatic progenitor cells [2, 5]. Thus, harnessing the regulatory mechanisms that control the proliferation and differentiation of liver progenitor cells may hold great promise to facilitating liver regeneration and/or progenitor cell-based therapies for liver diseases [5].

Several signaling pathways including Wnt signaling have been shown to play essential roles in liver development and postnatal liver regeneration [1, 2, 4, 5]. Wnt signaling plays essential roles in the development and numerous physiological and pathological processes [7–14]. Wnts are secreted glycoproteins that require extensive post-translation modifications for their biologic activity [15]. Biologically active Wnt ligands bind to receptor Frizzled (Fzd) and co-receptor LDL-related protein 5 (LRP5) or LRP6 to initiate a signaling cascade, which regulates a diverse set of downstream signaling events either through the canonical β-catenin/T-cell factor (TCF)/lymphoid enhancement factor (LEF) pathway, or noncanonical planar cell polarity (PCP) pathway or the Wnt/calcium pathway [7, 15, 16]. Canonical Wnt/β-catenin signaling has been shown to play important roles in regulating liver development and the proliferation and differentiation of liver progenitor cells, as well as in metabolic zonation [9, 15, 17]. However, it remains to be determined whether noncanonical Wnts play any significant roles in regulating liver development and regeneration.

In order to gain mechanistic insights into the regulatory circuitry governing the proliferation and differentiation of hepatic progenitor cells, we previously established a reversibly immortalized mouse hepatic progenitor cell line (iHPx), which retains long-term proliferative activity and yet possesses hepatic differentiation potential upon stimulated with appropriate differentiation cues [18–22]. Here, we comprehensively analyze the expression pattern of the essential components of both canonical and noncanonical Wnt signaling pathways at the different postnatal stages of mouse liver tissues and the iHPx cells, and find that the noncanonical Wnts Wnt4, Wnt5a, Wnt9b, Wnt10a, and Wnt10b are highly expressed concordantly with the high levels of canonical Wnts in the analyzed liver tissues. The noncanonical Wnts Wnt5a, Wnt9b, Wnt10a and Wnt10b are able to antagonize Wnt3a-induced β-catenin/TCF activity, reduce the stemness of the iHPx progenitor cells, and promote hepatic differentiation of liver progenitors *in vitro*. *In vivo* stem cell implantation assay demonstrates that Wnt5a, Wnt9b, Wnt10a and Wnt10b can inhibit cell proliferation and promote hepatic differentiation of the iHPx progenitor cells. Thus, our results strongly suggest that noncanonical Wnt signaling may play an important role in fine-tuning the Wnt/β-catenin signaling activity during liver development and regeneration.

## RESULTS

### Both canonical and noncanonical Wnt signaling components, as well as Wnt signaling modulators, are dynamically expressed at different stages of postnatal liver development

Although the biological roles of several canonical Wnts and β-catenin signaling in liver development and hepatocellular tumorigenesis have been extensively studied [15, 17], it remains to be fully understood about how canonical Wnt signaling is modulated under physiological and/or pathological conditions of the liver. Here, we sought to analyze the expression pattern of most essential components of canonical and noncanonical Wnt signaling during postnatal liver development. We collected total RNA from freshly obtained liver tissues of newborn (0 day), two-week-old (14D), one-month-old (28D), and six-month-old (180D) mice, and conducted TqPCR analysis of essential components of Wnt signaling.

**Figure 1:**
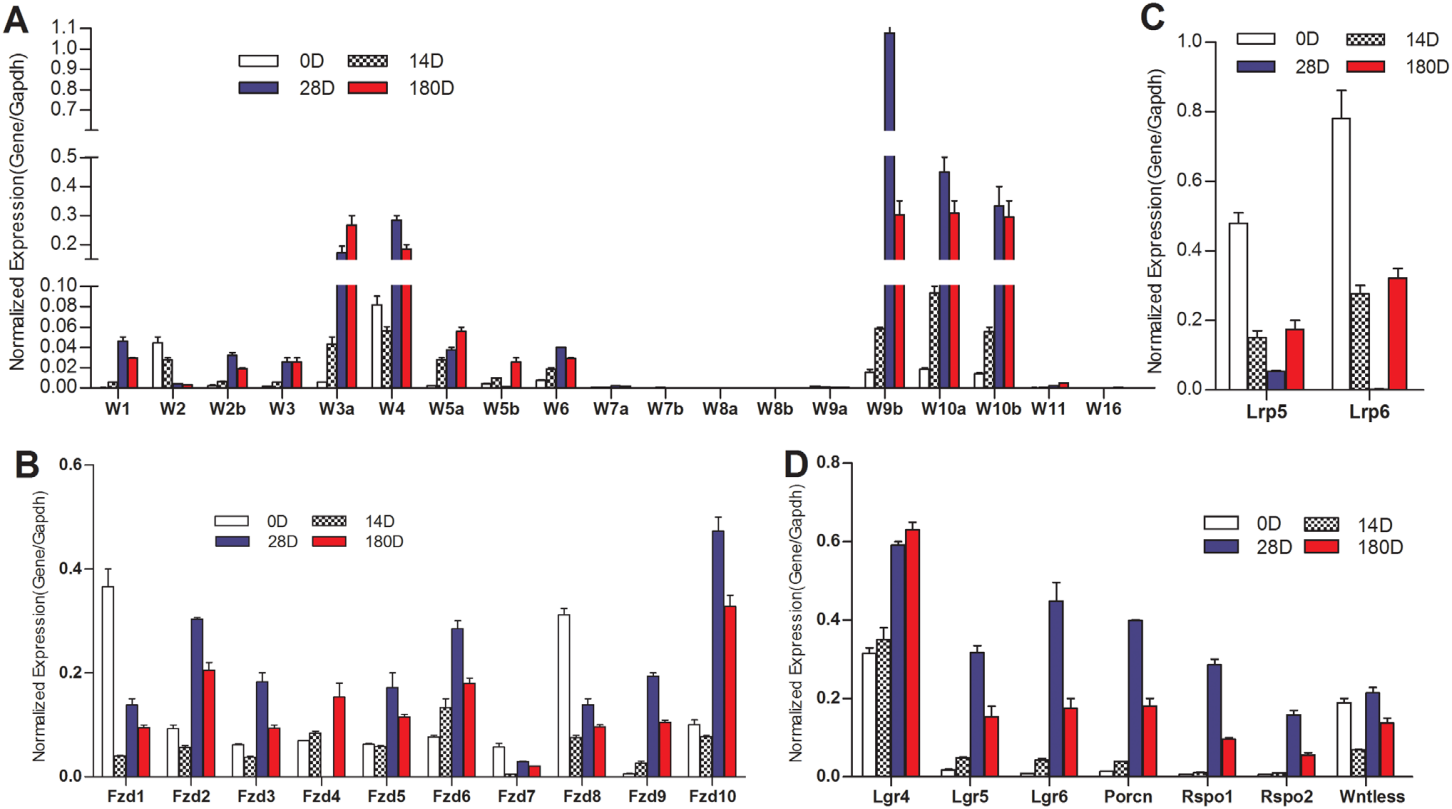
Expression patterns of the essential components of Wnt signaling pathway during mouse liver development Total RNA was isolated from mouse liver tissues at 0 (0D), 14 (14D), 28 (28D) and 180 (180D) days after birth. TqPCR analysis was carried out to detect the expression of 19 Wnts **(A)**, Wnt co-receptor LRP5 and LRP6 **(B)**, Wnt receptor Fzds **(C)**, and Wnt ligand/receptor-associated factors **(D)** All samples were normalized with Gapdh levels. Each assay condition was done in triplicate.

We first analyzed the expression profile of the 19 Wnt ligands in the liver samples collected from different time points. Our results indicate that the expression levels of seven of the 19 Wnts (i.e., Wnt7a, Wnt7b, Wnt8a, Wnt8b, Wnt9a, Wnt11 and Wnt16) were undetectable or very low under our assay condition (Figure 1A). The expression of five of the six canonical Wnts (e.g., Wnt1, Wnt2, Wnt3, Wnt3a and Wnt6) was readily detected and increased at later stages (D28 and D180) of liver development (Figure 1A). Noticeably, the commonly-studied canonical Wnt3a exhibited the highest expression levels, compared with other canonical Wnts, at late time points of liver development (Figure 1A). Surprisingly, several noncanical Wnts, such as Wnt2b, Wnt4, Wnt5a, Wnt5b, Wnt9b, Wnt10a and Wnt11, were highly expressed in 14D, 28D and 180D liver tissues. In particular, the expression levels of Wnt4, Wnt9b, Wnt10a and Wnt10b were at least equal to or higher than that of Wnt3a's at the same stages, and their expression levels were correlated with the development stages of liver (Figure 1A). These results showed that more noncanonical Wnts were highly expressed than canonical Wnts, suggesting that the expression of canonical Wnts may be counter-balanced by that of noncanonical Wnts, and that noncanonical Wnts may play an important role in modulating canonical Wnt signaling during postnatal liver development.

We further found that almost all of the ten Fzd receptors (except Fzd7) were highly expressed in four stages of liver samples, while Fzd2, Fzd6 and Fzd10 expressed highest levels in one-month and/or 6-month liver samples (Figure 1B). Interestingly, Fzd1 and Fzd8 expressed at the highest levels at birth (newborn) (Figure 1B). The expression levels of Wnt co-receptors Lrp5 and Lrp6 were readily detectable in most time points although shown at the highest at birth (Figure 1C). We further analyzed other factors that were associated with Wnt signaling, and found that Lgr4 and Wntless were highly expressed in all stages of liver samples, while Lgr5, Lgr6, Porcn, Rspo1, and Rspo2 were expressed at higher levels in one-month and 6-month liver samples (Figure 1D), suggesting that these accessory factors may express at comparable levels with the increased expression of Wnt ligands.

**Figure 2:**
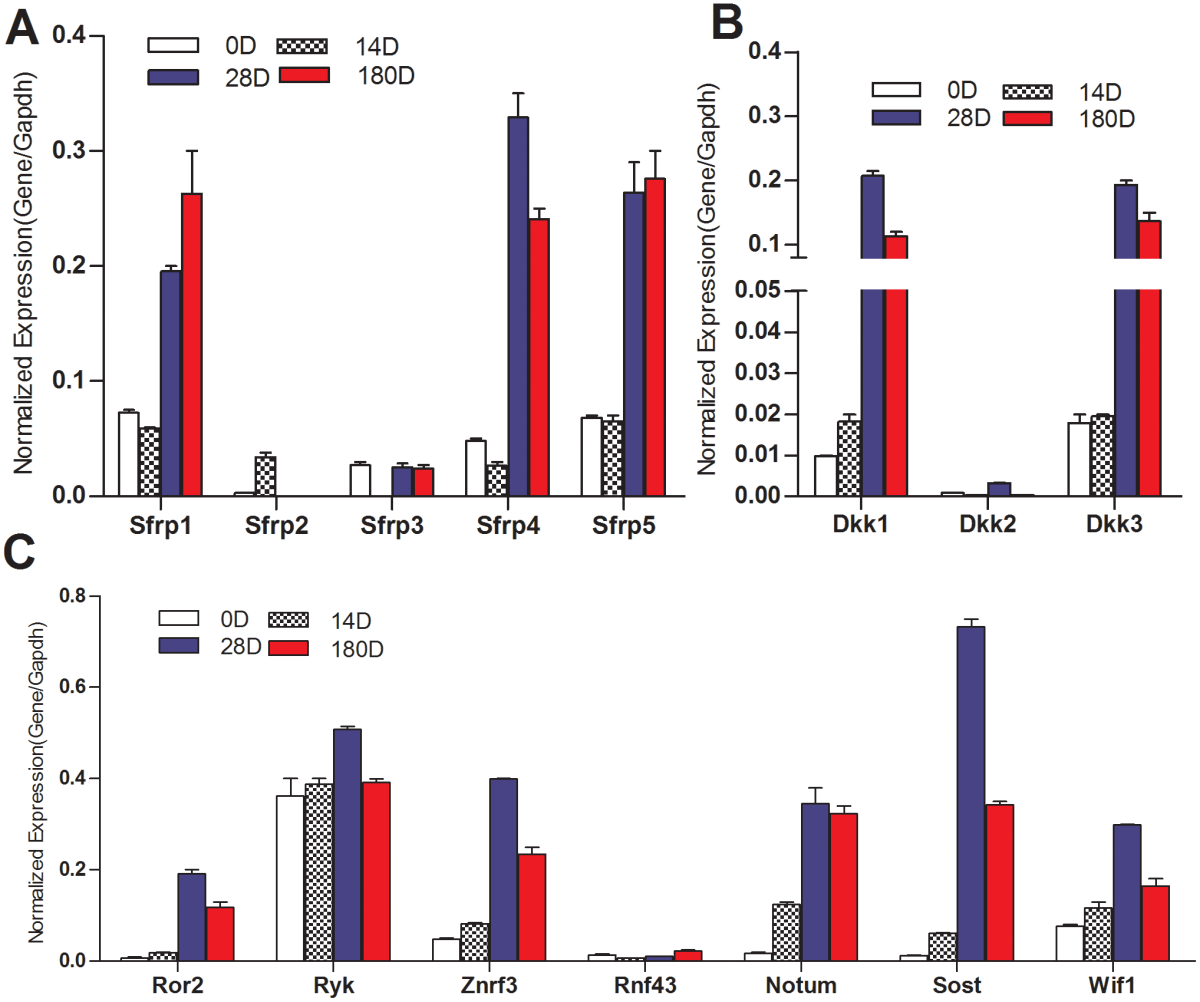
Expression patterns of the negative regulators of Wnt signaling pathway during mouse liver development Total RNA was isolated from mouse liver tissues as described in Figure 1. TqPCR analysis was performed to detect the expression of Fzd antagonists Sfrps **(A)**, Wnt co-receptor antagonists Dkks **(B)**, and several well-characterized negative regulators of Wnt signaling **(C)** All samples were normalized with Gapdh. Each assay condition was done in triplicate.

We further found that three of the five Fzd antagonists Sfrp1, Sfrp4, and Sfrp5 were highly expressed at late stages of liver development (e.g., 28D and 180D after birth), while Sfrp2 and Sfrp3 were expressed at low but detectable levels (Figure 2A). The Lrp5/6 antagonists Dkk1 and Dkk3 were also highly expressed at late stages of liver development (Figure 2B). The receptor-associated factor Ryk was highly expressed at all tested stages of liver development, while Ror2 was highly expressed in day 28 and 6 month liver samples (Figure 2C). Several negative regulators of Wnt signaling including Znrf3, Notum, Sost and Wif1, were highly expressed in later stages of liver development although Rnf43 was expressed at low but detectable levels (Figure 2C). These results strongly suggest that many of the Wnt negative regulators may be highly expressed during the differentiation and maturation of hepatocytes in order to maintain canonical Wnt signaling under fine-tuned regulation.

### The reversibly immortalized mouse liver progenitor iHPx cells serve as a good model cell system for studying canonical and noncanical Wnt signaling during hepatic differentiation

**Figure 3:**
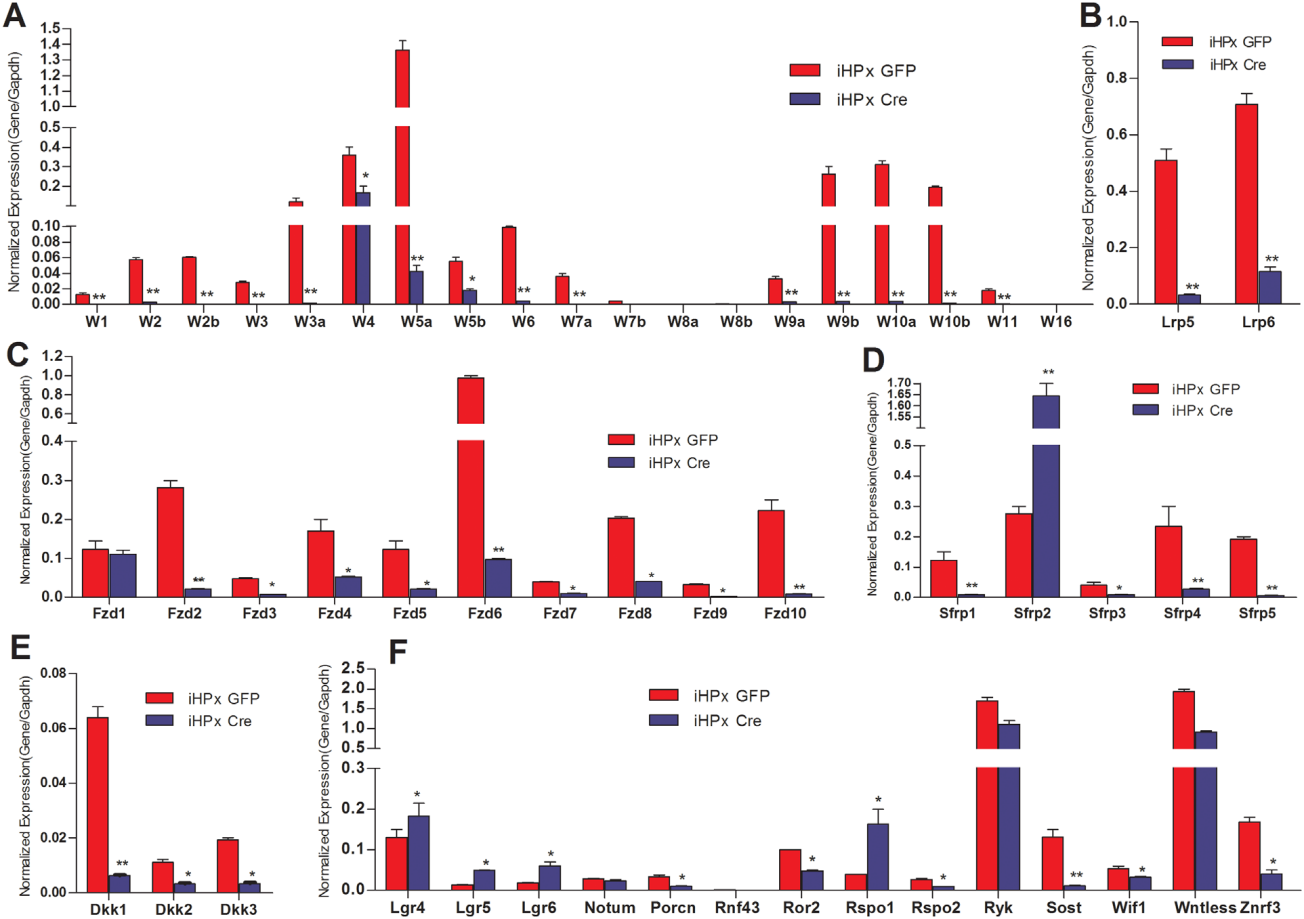
Expression patterns of the essential Wnt signaling components and regulators in the iHPx cells with or without the removal of the immortalizing gene SV40 T Antigen The reversibly immortalized iHPx cells were infected with Ad-GFP (iHPx GFP) or Ad-Cre (iHPx Cre) for 48h. Total RNA was isolated from the infected cells and subjected to reverse transcription. TqPCR analysis was carried out to detect the expression of the Wnt ligands **(A)**, co-receptors Lrp5 and 6 **(B)**, Wnt receptor Fzds **(C)**, Fzd antagonists Sfrps **(D)**, LRP co-receptor antagonists Dkks **(E)**, and other Wnt signaling regulators **(F)** All samples were normalized with Gapdh. Each assay condition was done in triplicate. “**” p < 0.001, “*” p < 0.05, Ad-GFP group vs. Ad-Cre group.

We previously established and characterized the reversibly immortalized mouse hepatic progenitor iHPx cells, which maintain the potential to differentiate into mature functional hepatocytes [18–22]. We next analyzed the expression patterns of Wnt ligands and their essential signaling components in the iHPx cells with or without the Cre-mediated removal of the immortalizing gene SV40 T antigen. We found the expression pattern of Wnt ligands in iHPx cells was very similar to that of the different stages of liver samples: the expression of five canonical Wnts (e.g., Wnt1, Wnt2, Wnt3, Wnt3a and Wnt7a) and 10 noncanonical Wnts (e.g., Wnt2, Wnt4, Wnt5a, Wnt5b, Wnt6, Wnt9a, Wnt9b, Wnt10a, Wnt10b and Wnt11) was readily detected (Figure 3A). The highly expressed Wnt ligands include Wnt3a, Wnt4, Wnt5a, Wnt9b, Wnt10a, and Wnt10b. These results reiterate the fact that more noncanonical Wnts were highly expressed than canonical Wnts in iHPx cells, further highlighting the check-balance of Wnt signaling in maintaining the stemness and differentiation of hepatic progenitor cells. However, a Cre-mediated removal of SV40 T antigen led to a pronounced decrease in the expression of both canonical and noncanonical Wnt. In particular, almost all canonical Wnts were expressed at extremely low; and only noncanonical Wnt4, Wnt5a and Wnt5b were expressed at relatively high levels (Figure 3A). We focused on four noncanonical Wnts (e.g., Wnt5a, Wnt9b, Wnt10a and Wnt10b) for further functional studies.

We further found that the expression patterns of co-receptors Lrp5 and Lrp6 and the Fzd receptors were similar to that of the 4-week old and 6-month-old liver samples’, and Fzd2, Fzd6, Fzd8 and Fzd10 were highly expressed in the iHPx cells (Figure 3B & 3C). However, upon the removal of SV40 T antigen from the iHPx cells, the expression levels of all co-receptors Lrp5 and Lrp6 and Fzds (except Fzd1) were significantly reduced (Figure 3B & 3C). It seems that Fzd1 expression was not affected by the removal of SV40 T antigen and remained as one of the highly expressed Fzds, along with Fzd4 and Fzd6 (Figure 3C). The expression of most Sfrps and Dkks were decreased upon the immortalization reversal of the iHPx cells (Figures 3D & 3E), although Sfrp2 expressed at a significantly higher level after the removal of SV40 T antigen (Figure 3D). Among other Wnt signaling regulators, we found that the expression of Porcn, Ror2, Ryk, Sost, Wif1, Wntless and Znrf3 was decreased upon the removal of SV40 T antigen, whereas the expression of Lgr4, Lgr5, Lgr6, and Rspo1was significantly increased when SV40 T antigen was removed (Figure 3F). These results demonstrate that the iHPx cells can be used as a model liver progenitor cells to study the effects of Wnt signaling on proliferation and differentiation. Furthermore, these results suggest that SV40 T antigen may immortalize the progenitors and preserve the stemness of the progenitors through up-regulating canonical Wnt signaling, which is counter-balanced by noncanonical Wnts and/or multiple negative regulators of Wnt signaling.

### The iHPx cells are responsive to canonical Wnt activation, which can be effectively antagonized by noncanonical Wnts

**Figure 4:**
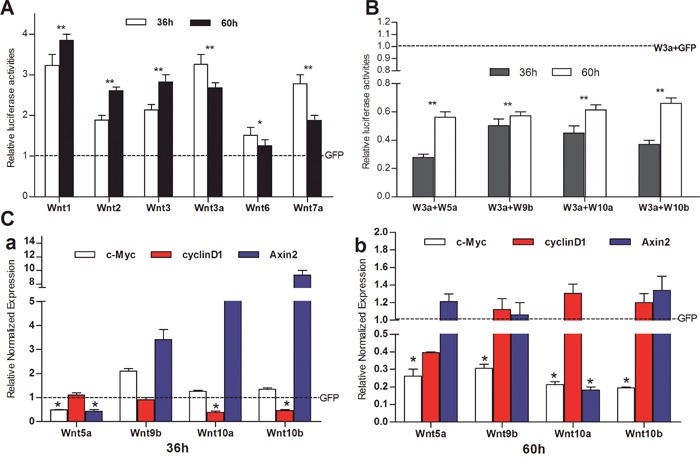
Non-canonical Wnts effectively inhibit the signaling activities of canonical Wnts in iHPx cells **A & B**. Subconfluent iHPx cells were infected with Ad-TOP-Luc for 16h, replated into 24-well cell culture plates, and infected with canonical Ad-Wnts or Ad-GFP (A), or co-infected with Ad-Wnt3a and non-canonical Wnts or Ad-GFP (B). At 36h and 60h after infection, cells were lysed and collected for luciferase activity assay using the Firefly Luciferase Assay Kit (Mirus Bio LLC, Madison, WI, USA). **C**. Subconfluent iHPx cells were infected with Ad-Wnt5a, Wnt9b, Wnt10a, Wnt10b, or Ad-GFP, respectively. At 36h (a) or 60h (b) after infection, total RNA was isolated and subjected to TqPCR analysis of the expression of canonical Wnt-regulated genes, Axin2, c-Myc, and cyclin D1. All samples were normalized with Gapdh. Each assay condition was done in triplicate. Relative expression was calculated by dividing the relative expression values (i.e., gene/Gapdh) in Wnt-treated group with that from the GFP-treated group. “**” p < 0.001, “*” p < 0.05, Ad-GFP group vs. Ad-Wnt group.

**Figure 5:**
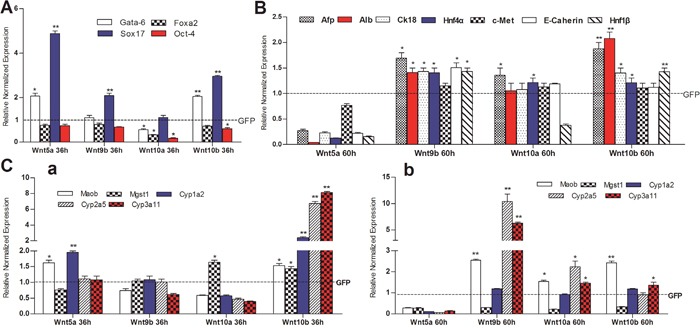
The effect of non-canonical Wnts on the expression of liver stem cell markers and hepatic differentiation-associated genes in the iHPx cells Subconfluent iHPx cells were infected with Ad-Wnt5a, Wnt9b, Wnt10a, Wnt10b, or Wnt-GFP for 36h (*a*) and 60h (*b*), respectively. Total RNA was isolated and subjected to TqPCR analysis of the expression of the liver stemness-related markers **(A)**, hepatic regulators and associated genes **(B)**, and mature hepatocyte markers **(C)** All samples were normalized with Gapdh. Each assay condition was done in triplicate. Relative expression was calculated by dividing the relative expression values (i.e., gene/Gapdh) in non-canonical Wnt-treated group with that from the GFP-treated group. “**” p < 0.001, “*” p < 0.05, Ad-GFP group vs. Ad-Wnt group.

To determine if the iHPx cells are responsive to canonical stimulation, we introduced the β-catenin/TCF4/LEF reporter TOP-Luc into the iHPx cells and transduced with adenoviral vectors expressing canonical Wnts, such as Wnt1, Wnt2, Wnt3, Wnt3a, Wnt6, Wnt7a, or GFP control for 36h and 60h. We found that the TOP-Luc reporter activities were significantly induced by all six tested canonical Wnts although Wnt6 was less effective in the iHPx cells (Figure 4A). To test potential direct inhibitory effect of noncanonical Wnts on canonical Wnts, we first introduced the TOP-Luc reporter into the iHPx cells, which were then co-infected with Wnt3a and noncanonical Wnts, such as Wnt5a, Wnt9b, Wnt10a, and Wnt10b, and found that Wnt3a-stimulated TOP-Luc activity was significantly inhibited by the tested four noncanonical Wnts (Figure 4B). We further examined the effect of noncanonical Wnts on the expression of canonical Wnt target genes Axin2, c-Myc and cyclin D1, and found that different noncanonical Wnts had distinct effects on the target gene expression (Figure 4C). Wnt5a inhibited Axin2 expression at 36h, while Wnt9b and Wnt10b significantly induced Axin2 expression (Figure 5C). However, Wnt9b, Wnt10a, and Wnt10b (especially Wnt10a) showed a trend to bring down the Axin2 levels to basal or lower levels (Figure 5C). While it's not clear why the noncanonical Wnts can up-regulate Axin2 expression, it's conceivable that such induction may be caused by feedback regulation of the canonical Wnt signaling pathway, particularly at the early stage of stimulations.

### Noncanonical Wnts inhibit the expression of hepatic stemness markers while induce the expression of hepatic differentiation associated markers

We further tested the effect of four noncanonical Wnts (e.g., Wnt5a, Wnt9b, Wnt10a and Wnt10b) on the expression of stemness markers and differentiation status of hepatic progenitors. We found that among the four noncanonical Wnts Wnt10a most effectively inhibited the expression of stem cell markers Gata-6, Foxa2 and Oct4, while no significant effect on Sox17 (Figure 5A). Wnt5a, Wnt9b and Wnt10b also effectively inhibited the expression of Foxa2 and Oct4, while inducing Sox17 as well Gata-6 to a lesser extent (Figure 5A). Furthermore, we analyzed the effect of the four noncanonical Wnts on the expression of hepatic regulator and liver marker genes such as Afp, Alb, Ck18, Hnf4α, c-Met, E-cadherin and Hnf1β, and found that Wnt9b, Wnt10a and Wnt10a effectively induced the expression of most if not all of these regulator and marker genes (Figure 5B). Interestingly, Wnt5a was shown to significantly inhibit the expression of Afp, Alb, Ck18, Hnf4α, c-Met, E-cadherin and Hnf1β (Figure 5B).

The effect of noncanonical Wnts on the expression of mature hepatocyte markers (e.g., Moab, Mgst1, Cyp1a2, Cyp2a5, and Cyp3a11) was also analyzed. We found that Wnt10b was able to induce the expression of these markers at 30h, but not at 60h (Figure 5C a *vs*. b). Wnt5a, Wnt9b and Wnt10a were shown to induce the expression of some but not all of the mature hepatocyte markers (Figure 5C *a & b*). We also analyzed the effect of noncanonical Wnts on the above markers in the Ad-Cre-treated iHPx cells and found the expression patterns were very similar (Supplementary Figure 1). Furthermore, using the previously established Alb-GLuc reporter line [18, 20, 21], we found that Wnt5a, Wnt9b, Wnt10a, and Wnt10b were able to induce GLuc activity significantly (data not shown).

**Figure 6:**
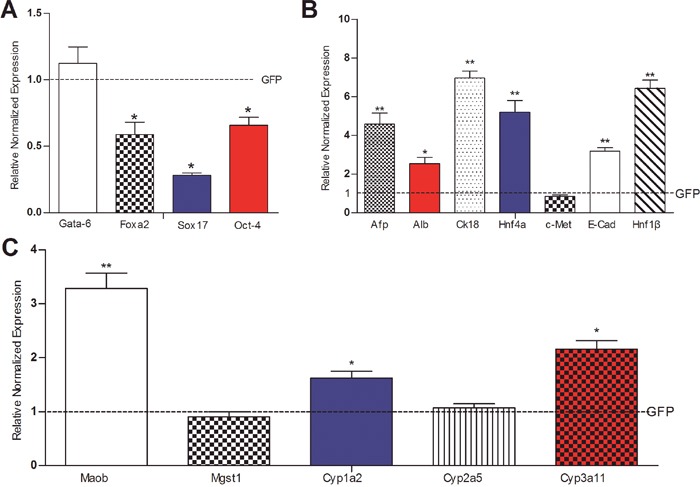
The effect of Cre-mediated removal of SV40 T antigen on the basal expression of liver stemness markers and hepatic differentiation-associated genes in the iHPx cells Subconfluent iHPx cells were infected with Ad-Cre or Ad-GFP for 48h. Total RNA was isolated and subjected to TqPCR analysis of the expression of the liver stemness-related markers **(A)**, hepatic regulators and associated genes **(B)**, and mature hepatocyte markers **(C)** All samples were normalized with Gapdh. Each assay condition was done in triplicate. Relative expression was calculated by dividing the relative expression values (i.e., gene/Gapdh) in non-canonical Wnt-treated group with that from the GFP-treated group. “**” p < 0.001, “*” p < 0.05, Ad-GFP group vs. Ad-Cre group.

Considering that the SV40 T antigen may affect cell proliferative activity and differentiation potential in the iHPx cells, we transduced the iHPx cells with Ad-Cre and examined the endogenous expression levels of the above stemness marker genes and hepatic regulator/marker genes. We demonstrated that the SV40 T antigen was effectively removed (data not shown), and found that the removal of SV40 T antigen led to a significant decrease in the expression of stem cell markers Foxa2, Sox17 and Oct-4 although Gata-6 expression was not affected (Figure 6A). However, the expression of Afp, Alb, Ck18, Hnf4α, E-cadherin, and Hnf1β, but not c-Met, was significantly increased upon the removal of SV40 T antigen (Figure 6). Furthermore, the expression of mature hepatocyte functional markers Maob, Cyp1a2, and Cyp3a11, but not Mgst1 and Cyp2a5, also increased significantly (Figure 6C). These results strongly suggest that the removal of the immortalizing SV40 T antigen in the iHPx cells may reduce the stenmess of the cells and facilitate terminal differentiation of iHPx cells, which is reminiscent of the noncanonical Wnts’ effect on liver progenitor cells.

### Noncanonical Wnts promote hepatic differentiation and facilitate hepatocyte maturation of the iHPx cells

**Figure 7:**
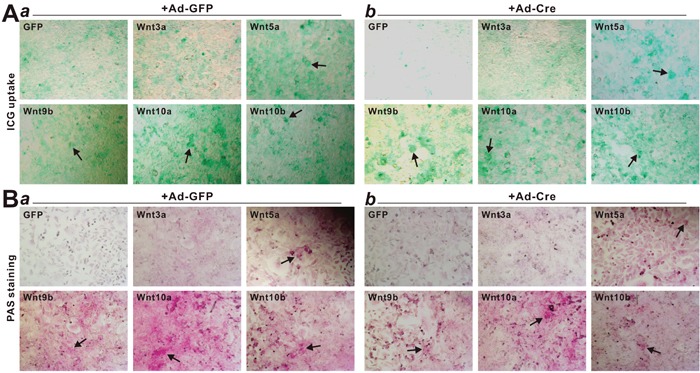
Non-canonical Wnts promote ICG uptake and glycogen synthesis/storage in iHPx cells **A**. NoncanonicalWnt-induced Indocyanine Green (ICG) uptake. Subconfluent iHPx cells were co-infected with Ad-GFP (*a*) or Ad-Cre (*b*) and the indicated Ad-Wnts for 10 days. Cells were washed with PBS and incubated with ICG (1mg/mL in complete DMEM) at 37°C for 1h, followed by PBS washes. ICG uptake was documented under a microscope. Each assay conditions were done in triplicate. Representative images are shown. Representative positive stains are indicated by arrows. **B**. NoncanonicalWnt-induced PAS staining for glycogen storage. Subconfluent iHPx cells were co-infected with Ad-GFP (*a*) or Ad-Cre (*b*) and the indicated Ad-Wnts for 10 days. Cells were fixed with 4% paraformaldehyde and stained with 0.5% periodic acid solution and the Schiff's reagent. Each assay condition was done in triplicate. Representative results are shown. Representative positive stains are indicated by arrows.

We also examined the effect of noncanonical Wnts on hepatic differentiation and maturation of iHPx cells by analyzing the ICG uptake and glycogen synthesis/storage assays. When the iHPx cells were stimulated with Wnt5a, Wnt9b, Wnt10a, Wnt10b, Wnt3a or GFP control, we found that three of the four noncanonical Wnts, Wnt5a, Wnt10a and Wnt11b, were shown to effectively promote ICG update, when compared to that of GFP control or Wnt3a, although the removal of SV40 T antigen only slightly enhanced the Wnt-induced ICG uptake (Figure 7A, *a vs. b*).

PAS staining is one of the commonly used assays to assess the glycogen synthesis and storage function of mature hepatocytes. We found that Wnt10a-stimulated iHPx cells exhibited most significant PAS-positive staining, while Wnt5a, Wnt9b, and Wnt10b were shown to induce a significantly higher level of PAS staining than that that for the GFP control or Wnt3a treated cells (Figure 7B). Similar to the ICG uptake assay, the PAS staining results were not significantly affected by the removal of SV40 T antigen (Figure 7B, a *vs*. b). Collectively, these results indicate that noncanonical Wnts (e.g., Wnt5a, Wnt9b, Wnt10a and Wnt10b) can promote hepatic differentiation of hepatic progenitors.

**Figure 8:**
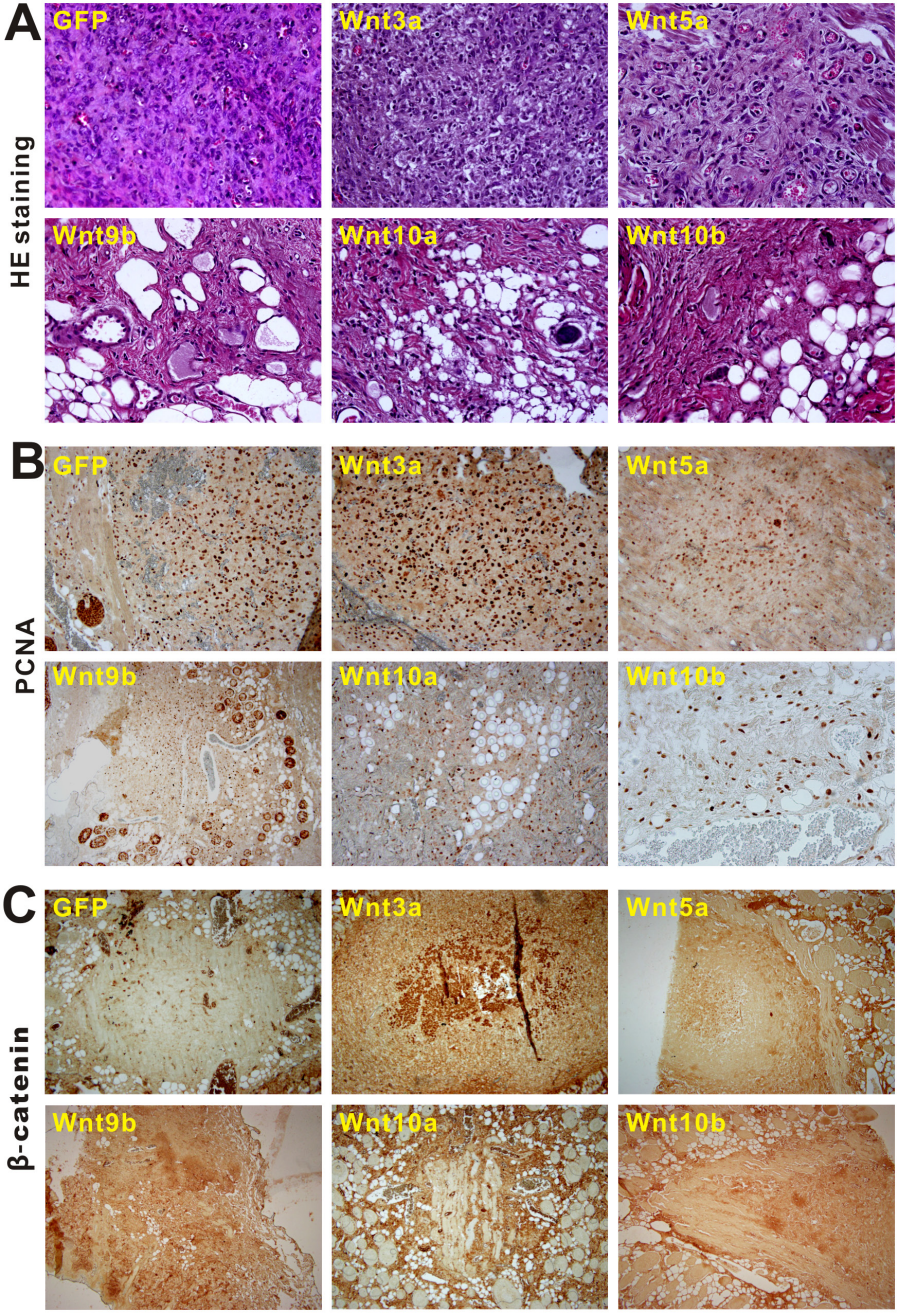
Nonecanonical Wnts induce cells differentiation, inhibit cell proliferation and downregulate β-catenin expression of the iHPx cells in the *in vivo* stem cell implantation assay Subconfluent iHPx cells were infected with Ad-Wnt3a, Ad-Wnt5a, Ad-Wnt9b, Ad-Wnt10a, Ad-Wnt10b, or Ad-GFP for 36h. The cells were collected and injected into athymic nude mice subcutaneously for 10 days. Masses at the injection sites were retrieved, fixed, paraffin-embedded, and subjected to H & E staining **(A)**. Sections were further subjected to immunohistochemical staining using either PCNA **(B)** or β-catenin **(C)** antibody (both from Santa Cruz Biotechnology). Control IgG and minus primary antibodies were used as negative controls (not shown). Representative images are shown.

Lastly, we tested the *in vivo* effect of noncanonical Wnts on the proliferation and β-catenin expression of the iHPx cells. Using the subcutaneous stem cell implantation assay [23–25], we found that the Ad-Wnt or Ad-GFP-transduced iHPx cells formed transient masses within the first two weeks, which gradually disappeared after two weeks. These results were consistent with our earlier findings in which the iHPx cells are not tumorigenic and rarely undergo spontaneous differentiation [18, 20–22]. We were able to recover cellular masses at 10 days post injection. Histological analysis indicated that, while Wnt3a promoted significant cell proliferation, Wnt5a, Wnt9b, Wnt10a and Wnt10b formed significantly smaller masses and inhibited cell proliferation, compared to that of the GFP-treated cells (Figure 8A). The cellular proliferative activity was further confirmed by PCNA immunohistochemical staining. Both Wnt3a and GFP-treated group exhibited high levels of PCNA-positive staining, while the masses retrieved from Wnt5a, Wnt9b, Wnt10a and Wnt10b groups had a significantly weaker or more sparsely positive staining (Figure 8B). Lastly, we examined the β-catenin status in these samples and found that, compared to the strongly positive staining in the Wnt3a treatment group, the masses recovered from Wnt5a, Wnt9b, Wnt10a and Wnt10b groups only exhibited weak or marginally positive β-catenin staining (Figure 8C), consistent with the *in vitro* results about the inhibitory effect of noncanonical Wnts on Wnt3a signaling activity in the iHPx cells

## DISCUSSION

Wnt signaling transduces evolutionarily conserved pathways which play important roles in initiating and regulating a diverse range of cellular activities, including cell proliferation, calcium homeostasis, and cell polarity [7–13]. Wnt signaling is a rather complex signaling network, which contains 19 Wnt ligands, 10 Fzd receptors, several types of co-receptors and numerous antagonists/negative regulators [12, 13]. Traditionally, Wnt signaling is classified into two large categories: the canonical Wnt (or β-catenin-dependent) and non-canonical Wnt (or β-catenin-independent) pathways [12, 13]. The canonical and non-canonical pathways form intersecting signaling networks that coordinately regulate complex processes, such as embryonic development, stem cell maintenance, tissue homeostasis, and wound healing [12, 13]. It's generally assumed that the canonical Wnt/β-catenin pathway regulates cell fate, proliferation, and survival, while non-canonical Wnt pathways are more often associated with differentiation, cell polarity, and migration [7–13]. Canonical Wnt/β-catenin signaling has been shown to play important roles in regulating the proliferation and differentiation of liver progenitor cells and metabolic zonation [9, 15, 17]. However, possible roles of noncanonical Wnts in regulating liver development and regeneration remain undefined.

**Figure 9:**
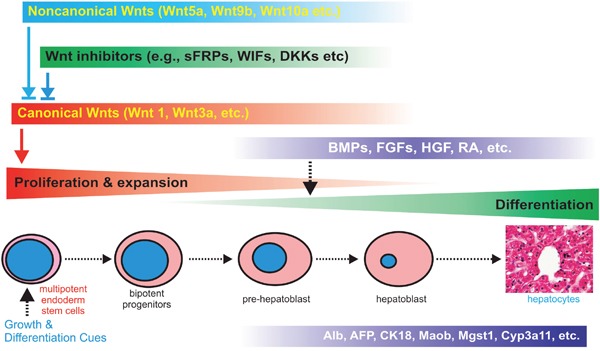
Nonecanonical Wnts modulates canonical Wnt-regulated cell proliferation and differentiation of liver progenitor cells Canonical Wnts promote the proliferation, stemness and expansion of hepatic progenitors, which is tightly controlled by negative regulators, including noncanonical Wnts and naturally occurring antagonists (e.g., sFRPs) and inhibitors (e.g., DKKs and WIFs). Hepatic differentiation may be further regulated by other signaling pathways, such as BMPs, FGFs and retinoic acid receptors.

In this study, we conduct a comprehensive analysis of the expression patterns of Wnt signaling components and find that several noncanonical Wnts, such as Wnt4, Wnt5a, Wnt9b, Wnt10a and Wnt10b, are highly expressed concordantly with the high levels of canonical Wnts in postnatal mouse liver tissues and the iHPx progenitor cells. Noncanonical Wnts Wnt5a, Wnt9b, Wnt10a and Wnt10b are able to antagonize Wnt3a-induced β-catenin/TCF reporter activity, reduce the stemness of the iHPx progenitor cells, and promote hepatic differentiation of liver progenitors *in vitro*. *In vivo* stem cell implantation assay demonstrates that Wnt5a, Wnt9b, Wnt10a and Wnt10b can inhibit cell proliferation and promote hepatic differentiation of the iHPx progenitor cells. Thus, our findings highlight the potentially critical roles of noncanonical Wnt signaling in maintaining a fine-tuned modulation of liver progenitor cells by canonical Wnt/β-catenin under physiological conditions (Figure 9).

Noncanonical Wnt signaling can be initiated by Wnt interaction with Frizzled receptors (Fzds), or RYK and ROR receptor tyrosine kinases and regulates small GTPases (such as RhoA, Rac, and Cdc42), and/or activates calcium flux and kinase cascades, including those of protein kinase C (PKC), calcium/calmodulin-dependent protein kinase II (CaMKII), and JUN N-terminal kinase (JNK) [7–13]. Interestingly, a recent study examined the important role of Wnt5a in hepatocyte proliferation and liver regeneration [26]. Using the liver-specific knockout models of Wntless (Wls-LKO), Yang et al showed that when subjected to partial hepatectomy the Wls-LKO showed prolongation of hepatocyte proliferation for up to 4 days compared with littermate controls, which coincided with increased β-catenin/TCF activity and decreased expression and secretion of Wnt5a [26]. However, Wnt5a expression increased between 24 and 48 hours, and Fzd2 between 24 and 72 hours after partial hepatectomy in normal mice, suggesting that Wnt5a may suppress β-catenin signaling in hepatocytes, thereby contributing to timely conclusion of the liver regeneration [26]. These findings are largely consistent with our results and further emphasize the important role of noncanonical Wnts in restricting Wnt/β-catenin signaling in liver development and liver regeneration.

Taken together, our findings highlight the potentially critical roles of noncanonical Wnt signaling in maintaining a fine-tuned modulation of liver progenitor cells by canonical Wnt/β-catenin under physiological conditions (Figure 9). It would be of significance to investigate the roles of canonical Wnts such as Wnt4, Wnt5a, Wnt9b, Wnt10a and Wnt10b in liver development and postnatal liver regeneration. Therefore, a thorough understanding of the potential functions of noncanonical Wnts in liver stem cells may unravel the mechanisms underlying the important roles of Wnt/β-catenin in liver development, liver regeneration, and the development of hepatocellular carcinoma.

## MATERIALS AND METHODS

### Cell culture and chemicals

HEK-293 derivative 293pTP cells were previously described [27]. The iHPx cells are the pooled mouse liver progenitors that were reversibly immortalized mouse E12.5 hepatic progenitor cells described previously [18, 20]. Both 293pTP and iHPx cells were maintained in complete Dulbecco's modified Eagle's medium (DMEM) supplemented with 10% fetal bovine serum (FBS, Gemini Bio-Products, West Sacramento, CA, USA), 100 units of penicillin and 100 mg of streptomycin at 37°C in 5% CO_2_. Unless indicated otherwise, all chemicals were purchased from Sigma-Aldrich (St Louis, MO, USA) or ThermoFisher Scientific (Waltham, MA, USA).

### Total RNA isolation and touchdown-quantitative real-time PCR (TqPCR) analysis

Total RNA from both cultured cells and fresh tissues was isolated by using the TRIZOL Reagent (ThermoFisher) according to the manufacturer's instructions. Briefly, the freshly prepared mouse liver tissues at the indicated development stages (n=5 CD1 male mice for each time point) were dissected out, quickly rinsed with PBS, minced, and then homogenized in the TRIzol reagent. For the cultured cells, subconfluent iHPx cells were treated with different conditions for varied duration and lysed in TRIZOL Reagent. Total RNA was extracted according to the manufacturer's instructions and subjected to reverse transcription reactions with hexamer and M-MuLV reverse transcriptase (New England Biolabs, Ipswich, MA). The cDNA products were further diluted and used as PCR templates. The gene-specific PCR primers were designed by using Primer3 program [28] (Supplementary Table 1). TqPCR was carried out by using SYBR Green-based TqPCR analysis on a CFX-Connect unit (Bio-Rad Laboratories, Hercules, CA) as described [23, 29, 30]. The TqPCR reactions were done in triplicate. Gapdh was used as a reference gene.

### Construction and amplification of recombinant adenoviruses Ad-Wnts, Ad-TOP-Luc, Ad-Cre, and Ad-GFP

Recombinant adenoviruses were generated using the AdEasy technology as described [31–34]. Briefly, the coding regions of mouse Wnt1, Wnt2, Wnt3, Wnt3a, Wnt5a, Wnt6, Wnt7a, Wnt9b, Wnt10a, and Wnt10b were PCR amplified and subcloned into an adenoviral shuttle vector, and subsequently used to generate recombinant adenoviruses in HEH-293 or 293pTP cells [27]. The resulting adenoviruses were designated as Ad-Wnt1, Ad-Wnt2, Ad-Wnt3, Ad-Wnt3a, Ad-Wnt5a, Ad-Wnt6, Ad-Wnt7a, Ad-Wnt9b, Ad-Wnt10a, and Ad-Wnt10b, all of which also express GFP. The recombinant adenovirus Ad-TOP-Luc containing the TCF/LEF-responsive elements driven firefly luciferase (FLuc) was also constructed and generated using the AdEasy technology [35]. The coding region of Cre recombinase was PCR amplified and subcloned into an adenoviral shuttle vector, and subsequently used to generate recombinant adenoviruses in 293pTP cells. An analogous adenovirus expressing GFP only (i.e., Ad-GFP) was used as a negative or mock virus control [36–39]. For all adenoviral infections, polybrene (4-8 μg/ml) was added to enhance infection efficiency as previously reported [40].

### TOP-Luc firefly luciferase (FLuc) reporter assay

The TOP-Luc reporter assays were carried out as described [41–44]. Briefly, exponentially growing iHPx cells were seeded in 100mm cell culture dishes and infected with Ad-TOP-Luc reporter virus for ∼16h, and then replated into 24-well plates, followed by infection with different Ad-Wnt viruses or Ad-GFP control. At 24h and 36h after infection, the cells were lysed and subjected to luciferase activity assays using the Firefly Luciferase Assay System (Promega, Madison, WI, USA). Each assay condition was done in triplicate.

### Indocyanine Green (ICG) uptake/release assay

The ICG uptake and release assay was carried out as described [18, 20, 21]. Briefly, exponentially growing iHPx cells were seeded in 24-well culture plates, and co-infected with Ad-GFP or Ad-Cre and Ad-Wnt3a, Ad-Wnt5a, Ad-Wnt9b, Ad-Wnt10a, or Ad-Wnt10b for 10 days. Cells were washed with PBS and incubated with ICG (1mg/mL in complete DMEM) at 37°C for 1h, followed by PBS washes. ICG uptake was observed and recorded under a bright field microscope. For ICG release detection, the ICG-containing medium was changed with complete DMEM, and the cells were incubated for additional 6h. The status of ICG-stained cells was again observed and recorded under a microscope. Each assay condition was done in triplicate.

### Periodic acid-schiff (PAS) staining

PAS staining was carried out as described [18, 20, 21]. Briefly, exponentially growing iHPx cells were seeded in 24-well culture plates and co-infected with Ad-GFP or Ad-Cre and Ad-Wnt5a, Ad-Wnt9b, Ad-Wnt10a, or Ad-Wnt10b. At 10 days post infection, cells were fixed with 4% paraformaldehyde for 10min, followed by stained with 0.5% periodic acid solution for 5min. After being rinsed in distilled water for 3 min, cells were incubated in the Schiff's reagent for 15min, followed by thorough rinses with tap water. Cell staining was recorded using a microscope. Each assay condition was done in triplicate.

### Subcutaneous stem cell implantation of iHPx cells

All animal experiments were carried out in accordance with the approved guidelines approved by the Institutional Animal Care and Use Committee. The stem cell implantation assays were carried out as described [45–49]. Briefly, the iHPx cells were infected with Ad-Wnt3a, Ad-Wnt5a, Ad-Wnt9b, Ad-Wnt10a, Ad-Wnt10b, Ad-GFP for 36h, and then collected and resuspended in PBS at 10^7^ cells/ml for subcutaneous injection into the flanks of athymic nude mice (Harlan Laboratories, 5-6 week old, male, 5×10^6^ cells/injection, 6 injections per mouse, and 5 mice per group). At 10 days after injection, mice were sacrificed, and subcutaneous masses were retrieved for histologic evaluation.

### H & E staining and immunohistochemical (IHC) staining of the masses retrieved from subcutaneous stem cells implantation

The retrieved tumor masses were fixed in formalin, paraffin-embedded, and sectioned. The slides were deparaffinized, rehydrated and subjected to H & E staining [50–52]. IHC staining was performed as described [53–55]. Briefly, the sections were subjected to deparaffinization, followed by antigen retrieval and immunostaining with an anti-PCNA or anti-β-catenin antibody (Santa Cruz Biotechnology). Control IgG and minus primary antibodies were used as negative controls.

### Statistical analysis

Quantitative data were expressed as mean ± standard deviation. Statistical analysis was carried out using Microsoft Excel program. Data were expressed as mean ± SD. Statistical significances were determined by one-way analysis of variance and the student's t test. A *p<* 0.05 was considered statistically significant.

## SUPPLEMENTARY FIGURE AND TABLE





## References

[1] Zaret KS (2008). Genetic programming of liver and pancreas progenitors: lessons for stem-cell differentiation. Nat Rev Genet.

[2] Kopp JL, Grompe M, Sander M (2016). Stem cells versus plasticity in liver and pancreas regeneration. Nat Cell Biol.

[3] Si-Tayeb K, Lemaigre FP, Duncan SA (2010). Organogenesis and development of the liver. Dev Cell.

[4] Miyajima A, Tanaka M, Itoh T (2014). Stem/progenitor cells in liver development, homeostasis, regeneration, and reprogramming. Cell Stem Cell.

[5] Stanger BZ (2015). Cellular homeostasis and repair in the mammalian liver. Annu Rev Physiol.

[6] Geerts A (2004). On the origin of stellate cells: mesodermal, endodermal or neuro-ectodermal?. J Hepatol.

[7] Nusse R (2005). Wnt signaling in disease and in development. Cell Res.

[8] Nusse R, Fuerer C, Ching W, Harnish K, Logan C, Zeng A, ten Berge D, Kalani Y (2008). Wnt signaling and stem cell control. Cold Spring Harb Symp Quant Biol.

[9] Clevers H, Loh KM, Nusse R (2014). Stem cell signaling. An integral program for tissue renewal and regeneration: wnt signaling and stem cell control. Science.

[10] Clevers H, Nusse R (2012). Wnt/β-catenin signaling and disease. Cell.

[11] Kim JH, Liu X, Wang J, Chen X, Zhang H, Kim SH, Cui J, Li R, Zhang W, Kong Y, Zhang J, Shui W, Lamplot J (2013). Wnt signaling in bone formation and its therapeutic potential for bone diseases. Ther Adv Musculoskelet Dis.

[12] Mohammed MK, Shao C, Wang J, Wei Q, Wang X, Collier Z, Tang S, Liu H, Zhang F, Huang J, Guo D, Lu M, Liu F (2016). Wnt/β-catenin signaling plays an ever-expanding role in stem cell self-renewal, tumorigenesis and cancer chemoresistance. Genes Dis.

[13] Yang K, Wang X, Zhang H, Wang Z, Nan G, Li Y, Zhang F, Mohammed MK, Haydon RC, Luu HH, Bi Y, He TC (2016). The evolving roles of canonical WNT signaling in stem cells and tumorigenesis: implications in targeted cancer therapies. Lab Invest.

[14] Zhang F, Song J, Zhang H, Huang E, Song D, Tollemar V, Wang J, Wang J, Mohammed M, Wei Q, Fan J, Liao L, Zou Y (2016). Wnt and BMP signaling crosstalk in regulating dental stem cells: implications in dental tissue engineering. Genes Dis.

[15] Monga SP (2015). β-Catenin Signaling and Roles in Liver Homeostasis, Injury, and Tumorigenesis. Gastroenterology.

[16] Luo J, Chen J, Deng ZL, Luo X, Song WX, Sharff KA, Tang N, Haydon RC, Luu HH, He TC (2007). Wnt signaling and human diseases: what are the therapeutic implications?. Lab Invest.

[17] Monga SP (2011). Role of Wnt/β-catenin signaling in liver metabolism and cancer. Int J Biochem Cell Biol.

[18] Bi Y, He Y, Huang J, Su Y, Zhu GH, Wang Y, Qiao M, Zhang BQ, Zhang H, Wang Z, Liu W, Cui J, Kang Q (2014). Functional characteristics of reversibly immortalized hepatic progenitor cells derived from mouse embryonic liver. Cell Physiol Biochem.

[19] Bi Y, He Y, Huang JY, Xu L, Tang N, He TC, Feng T (2013). Induced maturation of hepatic progenitor cells *in vitro*. Braz J Med Biol Res.

[20] Bi Y, Huang J, He Y, Zhu GH, Su Y, He BC, Luo J, Wang Y, Kang Q, Luo Q, Chen L, Zuo GW, Jiang W (2009). Wnt antagonist SFRP3 inhibits the differentiation of mouse hepatic progenitor cells. J Cell Biochem.

[21] Huang J, Bi Y, Zhu GH, He Y, Su Y, He BC, Wang Y, Kang Q, Chen L, Zuo GW, Luo Q, Shi Q, Zhang BQ (2009). Retinoic acid signalling induces the differentiation of mouse fetal liver-derived hepatic progenitor cells. Liver Int.

[22] Wang X, Cui J, Zhang BQ, Zhang H, Bi Y, Kang Q, Wang N, Bie P, Yang Z, Wang H, Liu X, Haydon RC, Luu HH (2014). Decellularized liver scaffolds effectively support the proliferation and differentiation of mouse fetal hepatic progenitors. J Biomed Mater Res A.

[23] Lu S, Wang J, Ye J, Zou Y, Zhu Y, Wei Q, Wang X, Tang S, Liu H, Fan J, Zhang F, Farina EM, Mohammed MM (2016). Bone morphogenetic protein 9 (BMP9) induces effective bone formation from reversibly immortalized multipotent adipose-derived (iMAD) mesenchymal stem cells. Am J Transl Res.

[24] Ye J, Wang J, Zhu Y, Wei Q, Wang X, Yang J, Tang S, Liu H, Fan J, Zhang F, Farina EM, Mohammed MK, Zou Y (2016). A thermoresponsive polydiolcitrate-gelatin scaffold and delivery system mediates effective bone formation from BMP9-transduced mesenchymal stem cells. Biomed Mater.

[25] Deng F, Chen X, Liao Z, Yan Z, Wang Z, Deng Y, Zhang Q, Zhang Z, Ye J, Qiao M, Li R, Denduluri S, Wang J (2014). A simplified and versatile system for the simultaneous expression of multiple siRNAs in mammalian cells using Gibson DNA Assembly. PLoS One.

[26] Yang J, Cusimano A, Monga JK, Preziosi ME, Pullara F, Calero G, Lang R, Yamaguchi TP, Nejak-Bowen KN, Monga SP (2015). WNT5A inhibits hepatocyte proliferation and concludes β-catenin signaling in liver regeneration. Am J Pathol.

[27] Wu N, Zhang H, Deng F, Li R, Zhang W, Chen X, Wen S, Wang N, Zhang J, Yin L, Liao Z, Zhang Z, Zhang Q (2014). Overexpression of Ad5 precursor terminal protein accelerates recombinant adenovirus packaging and amplification in HEK-293 packaging cells. Gene Ther.

[28] Untergasser A, Cutcutache I, Koressaar T, Ye J, Faircloth BC, Remm M, Rozen SG (2012). Primer3—new capabilities and interfaces. Nucleic Acids Res.

[29] Zhang Q, Wang J, Deng F, Yan Z, Xia Y, Wang Z, Ye J, Deng Y, Zhang Z, Qiao M, Li R, Denduluri SK, Wei Q (2015). TqPCR: A Touchdown qPCR Assay with Significantly Improved Detection Sensitivity and Amplification Efficiency of SYBR Green qPCR. PLoS One.

[30] Denduluri SK, Scott B, Lamplot JD, Yin L, Yan Z, Wang Z, Ye J, Wang J, Wei Q, Mohammed MK, Haydon RC, Kang RW, He TC (2016). Immortalized Mouse Achilles Tenocytes Demonstrate Long-Term Proliferative Capacity While Retaining Tenogenic Properties. Tissue Eng Part C Methods.

[31] He TC (2004). Adenoviral Vectors. Adenoviral Vectors in Current Protocols in Human Genetics.

[32] He TC, Zhou S, da Costa LT, Yu J, Kinzler KW, Vogelstein B (1998). A simplified system for generating recombinant adenoviruses. Proc Natl Acad Sci USA.

[33] Breyer B, Jiang W, Cheng H, Zhou L, Paul R, Feng T, He TC (2001). Adenoviral vector-mediated gene transfer for human gene therapy. Curr Gene Ther.

[34] Luo J, Deng ZL, Luo X, Tang N, Song WX, Chen J, Sharff KA, Luu HH, Haydon RC, Kinzler KW, Vogelstein B, He TC (2007). A protocol for rapid generation of recombinant adenoviruses using the AdEasy system. Nat Protoc.

[35] Zhang F, Li Y, Zhang H, Huang E, Gao L, Luo W, Wei Q, Fan J, Song D, Liao J, Zou Y, Liu F, Liu J (2017). Anthelmintic mebendazole enhances cisplatin's effect on suppressing cell proliferation and promotes differentiation of head and neck squamous cell carcinoma (HNSCC). Oncotarget.

[36] Zhang H, Wang J, Deng F, Huang E, Yan Z, Wang Z, Deng Y, Zhang Q, Zhang Z, Ye J, Qiao M, Li R, Wang J (2015). Canonical Wnt signaling acts synergistically on BMP9-induced osteo/odontoblastic differentiation of stem cells of dental apical papilla (SCAPs). Biomaterials.

[37] Deng Y, Wang Z, Zhang F, Qiao M, Yan Z, Wei Q, Wang J, Liu H, Fan J, Zou Y, Liao J, Hu X, Chen L (2016). A Blockade of IGF Signaling Sensitizes Human Ovarian Cancer Cells to the Anthelmintic Niclosamide-Induced Anti-Proliferative and Anticancer Activities. Cell Physiol Biochem.

[38] Deng Y, Zhang J, Wang Z, Yan Z, Qiao M, Ye J, Wei Q, Wang J, Wang X, Zhao L, Lu S, Tang S, Mohammed MK (2015). Antibiotic monensin synergizes with EGFR inhibitors and oxaliplatin to suppress the proliferation of human ovarian cancer cells. Sci Rep.

[39] Li R, Yan Z, Ye J, Huang H, Wang Z, Wei Q, Wang J, Zhao L, Lu S, Wang X, Tang S, Fan J, Zhang F (2016). The Prodomain-Containing BMP9 Produced from a Stable Line Effectively Regulates the Differentiation of Mesenchymal Stem Cells. Int J Med Sci.

[40] Zhao C, Wu N, Deng F, Zhang H, Wang N, Zhang W, Chen X, Wen S, Zhang J, Yin L, Liao Z, Zhang Z, Zhang Q (2014). Adenovirus-mediated gene transfer in mesenchymal stem cells can be significantly enhanced by the cationic polymer polybrene. PLoS One.

[41] Luo Q, Kang Q, Si W, Jiang W, Park JK, Peng Y, Li X, Luu HH, Luo J, Montag AG, Haydon RC, He TC (2004). Connective tissue growth factor (CTGF) is regulated by Wnt and bone morphogenetic proteins signaling in osteoblast differentiation of mesenchymal stem cells. J Biol Chem.

[42] Tang N, Song WX, Luo J, Luo X, Chen J, Sharff KA, Bi Y, He BC, Huang JY, Zhu GH, Su YX, Jiang W, Tang M (2009). BMP-9-induced osteogenic differentiation of mesenchymal progenitors requires functional canonical Wnt/beta-catenin signalling. J Cell Mol Med.

[43] Si W, Kang Q, Luu HH, Park JK, Luo Q, Song WX, Jiang W, Luo X, Li X, Yin H, Montag AG, Haydon RC, He TC (2006). CCN1/Cyr61 is regulated by the canonical Wnt signal and plays an important role in Wnt3A-induced osteoblast differentiation of mesenchymal stem cells. Mol Cell Biol.

[44] He BC, Gao JL, Zhang BQ, Luo Q, Shi Q, Kim SH, Huang E, Gao Y, Yang K, Wagner ER, Wang L, Tang N, Luo J (2011). Tetrandrine inhibits Wnt/β-catenin signaling and suppresses tumor growth of human colorectal cancer. Mol Pharmacol.

[45] Huang E, Bi Y, Jiang W, Luo X, Yang K, Gao JL, Gao Y, Luo Q, Shi Q, Kim SH, Liu X, Li M, Hu N (2012). Conditionally immortalized mouse embryonic fibroblasts retain proliferative activity without compromising multipotent differentiation potential. PLoS One.

[46] Gao Y, Huang E, Zhang H, Wang J, Wu N, Chen X, Wang N, Wen S, Nan G, Deng F, Liao Z, Wu D, Zhang B (2013). Crosstalk between Wnt/β-catenin and estrogen receptor signaling synergistically promotes osteogenic differentiation of mesenchymal progenitor cells. PLoS One.

[47] Lamplot JD, Liu B, Yin L, Zhang W, Wang Z, Luther G, Wagner E, Li R, Nan G, Shui W, Yan Z, Rames R, Deng F (2015). Reversibly Immortalized Mouse Articular Chondrocytes Acquire Long-Term Proliferative Capability While Retaining Chondrogenic Phenotype. Cell Transplant.

[48] Wang N, Zhang W, Cui J, Zhang H, Chen X, Li R, Wu N, Chen X, Wen S, Zhang J, Yin L, Deng F, Liao Z (2014). The piggyBac transposon-mediated expression of SV40 T antigen efficiently immortalizes mouse embryonic fibroblasts (MEFs). PLoS One.

[49] Wang J, Zhang H, Zhang W, Huang E, Wang N, Wu N, Wen S, Chen X, Liao Z, Deng F, Yin L, Zhang J, Zhang Q (2014). Bone morphogenetic protein-9 effectively induces osteo/odontoblastic differentiation of the reversibly immortalized stem cells of dental apical papilla. Stem Cells Dev.

[50] Liao Z, Nan G, Yan Z, Zeng L, Deng Y, Ye J, Zhang Z, Qiao M, Li R, Denduluri S, Wang J, Wei Q, Geng N (2015). The Anthelmintic Drug Niclosamide Inhibits the Proliferative Activity of Human Osteosarcoma Cells by Targeting Multiple Signal Pathways. Curr Cancer Drug Targets.

[51] Yan Z, Yin L, Wang Z, Ye J, Zhang Z, Li R, Denduluri SK, Wang J, Wei Q, Zhao L, Lu S, Wang X, Tang S (2016). A Novel Organ Culture Model of Mouse Intervertebral Disc Tissues. Cells Tissues Organs.

[52] Li R, Zhang W, Cui J, Shui W, Yin L, Wang Y, Zhang H, Wang N, Wu N, Nan G, Chen X, Wen S, Deng F (2014). Targeting BMP9-promoted human osteosarcoma growth by inactivation of notch signaling. Curr Cancer Drug Targets.

[53] Chen X, Luther G, Zhang W, Nan G, Wagner ER, Liao Z, Wu N, Zhang H, Wang N, Wen S, He Y, Deng F, Zhang J (2013). The E-F hand calcium-binding protein S100A4 regulates the proliferation, survival and differentiation potential of human osteosarcoma cells. Cell Physiol Biochem.

[54] Hu N, Jiang D, Huang E, Liu X, Li R, Liang X, Kim SH, Chen X, Gao JL, Zhang H, Zhang W, Kong YH, Zhang J (2013). BMP9-regulated angiogenic signaling plays an important role in the osteogenic differentiation of mesenchymal progenitor cells. J Cell Sci.

[55] Huang E, Zhu G, Jiang W, Yang K, Gao Y, Luo Q, Gao JL, Kim SH, Liu X, Li M, Shi Q, Hu N, Wang L (2012). Growth hormone synergizes with BMP9 in osteogenic differentiation by activating the JAK/STAT/IGF1 pathway in murine multilineage cells. J Bone Miner Res.

