# Supplementary material for: Noncanonical Wnt signaling plays an important role in modulating canonical Wnt-regulated stemness, proliferation and terminal differentiation of hepatic progenitors
Source: Oncotarget. 2017 Feb 23;8(16):27105–19. doi: 10.18632/oncotarget.15637 (PMC5432321; doi:10.18632/oncotarget.15637)
Supplement: Supplementary file 1 [file oncotarget-08-27105-s001.pdf]

Noncanonical Wnt signaling plays an important role in modulating canonical Wnt-regulated stemness, proliferation and terminal differentiation of hepatic progenitors

SUPPLEMENTARY FIGURE AND TABLE

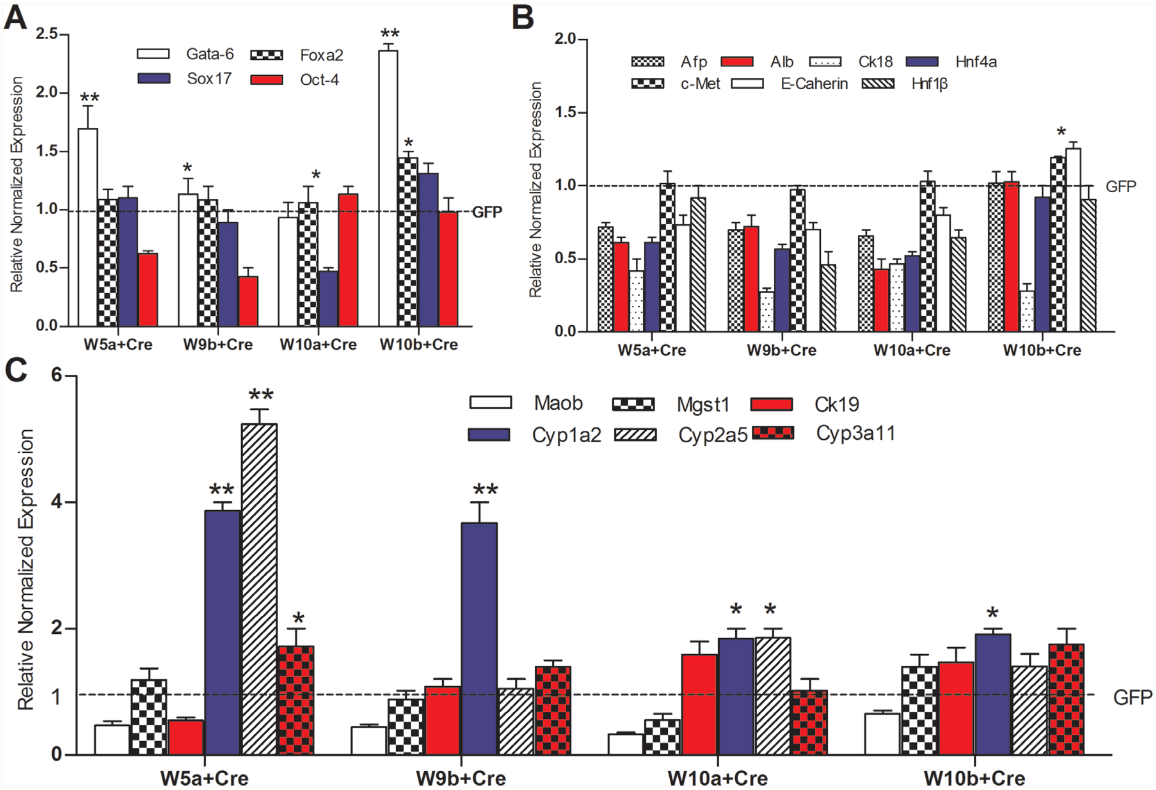

Supplementary Figure 1: The effect of non-canonical Wnts on the expression of liver stem cell markers and hepatic differentiation-associated genes in the iHPx cells after the removal of SV40 T antigen. Subconfluent iHPx cells were first infected with Ad-Cre or Ad-GFP for 24h, and then Ad-Wnt5a, Wnt9b, Wnt10a, or Wnt10b for additional 48h. Total RNA was isolated and subjected to TqPCR analysis of the expression of the liver stemness-related markers **(A)**, hepatic regulators and associated genes **(B)**, and mature hepatocyte markers **(C)** All samples were normalized with Gapdh. Each assay condition was done in triplicate. Relative expression was calculated by dividing the relative expression values (i.e., gene/Gapdh) in non-canonical Wnt-treated group with that from the GFP-treated group. "**" $p < 0.001$, "*" $p < 0.05$, Ad-GFP group vs. Ad-Wnt group.

Supplementary Table 1: List of TqPCR Primers

See Supplementary File 1
